# A survey of substance use for cognitive enhancement by university students in the Netherlands

**DOI:** 10.3389/fnsys.2015.00010

**Published:** 2015-02-17

**Authors:** Kimberly J. Schelle, Bas M. J. Olthof, Wesley Reintjes, Carsten Bundt, Joyce Gusman-Vermeer, Anke C. C. M. van Mil

**Affiliations:** ^1^Radboud Honours Academy, Radboud UniversityNijmegen, Netherlands; ^2^Department of Industrial Design, Eindhoven University of TechnologyEindhoven, Netherlands; ^3^Faculty of Medical Sciences, Institute of Neuroscience, Newcastle UniversityNewcastle upon Tyne, UK; ^4^Department of Neurology, Isala ClinicsZwolle, Netherlands; ^5^Department of Experimental Psychology, Ghent UniversityGhent, Belgium; ^6^Department of Physiology, Radboud Institute for Health Sciences, Radboud University Medical CenterNijmegen, Netherlands

**Keywords:** cognitive enhancement, neuroenhancement, smart drugs, prescription stimulants, non-medical use, illicit drugs, lifestyle drugs, self-report

## Abstract

**Background**: Pharmacological cognitive enhancement, using chemicals to change cellular processes in the brain in order to enhance one's cognitive capacities, is an often discussed phenomenon. The prevalence among Dutch university students is unknown.

**Methods:** The study set out to achieve the following goals: (1) give an overview of different methods in order to assess the prevalence of use of prescription, illicit and lifestyle drugs for cognitive enhancement (2) investigate whether polydrug use and stress have a relationship with cognitive enhancement substance use (3) assessing opinions about cognitive enhancement prescription drug use. A nationwide survey was conducted among 1572 student respondents of all government supported Dutch universities.

**Results:** The most detailed level of analysis—use of specific substances without a prescription and with the intention of cognitive enhancement—shows that prescription drugs, illicit drugs and lifestyle drugs are respectively used by 1.7, 1.3, and 45.6% of the sample. The use of prescription drugs and illicit drugs is low compared to other countries. We have found evidence of polydrug use in relation to cognitive enhancement. A relation between stress and the use of lifestyle drugs for cognitive enhancement was observed. We report the findings of several operationalizations of cognitive enhancement drug use to enable comparison with a wider variety of previous and upcoming research.

**Conclusions:** Results of this first study among university students in the Netherlands revealed a low prevalence of cognitive enhancement drug use compared to other countries. Multiple explanations, such as a difference in awareness of pharmacological cognitive enhancement among students, accessibility of drugs in the student population and inclusion criteria of enhancement substances are discussed. We urge enhancement researchers to take the different operationalizations and their effects on the prevalence numbers into account.

## Introduction

Recently the topic of cognitive enhancement has received much attention, both in popular media (e.g., Schwarz, 2012; Sharrett, 2012; Shiner, 2013; Monks, 2014) and academic literature (e.g., a special issue on cognitive enhancers by Neuropharmacology Lynch et al., 2013). Cognitive enhancement can be accomplished in a variety of ways. Some methods use advanced technology to modulate brain activity, e.g., deep brain stimulation, whilst other methods are based on chemicals that are aimed at changing the cellular processes in the brain, also named pharmacological cognitive enhancement. Pharmacological cognitive enhancement refers to boosting cognitive capacities by using substances, with the aim to enhance one's performance above baseline levels (e.g., Bostrom and Sandberg, 2009). Most substances regularly referred to in the discussion about pharmacological cognitive enhancement were originally developed to treat neuropsychiatric disorders that are often accompanied by cognitive deficits. A drug widely reported to be used with the purpose of pharmacological cognitive enhancement is methylphenidate (e.g., Ritalin, Concerta), which was developed as a treatment for attention deficit hyperactivity disorder (ADHD). Other drugs often used to boost cognitive capacities include modafinil and amphetamines (Ragan et al., 2013). However, recently also other substances, such as caffeine and nicotine as well as several illicit drugs, are included in the discussion of the cognitive enhancement literature (e.g., Franke et al., 2011b, 2012; Wolff et al., 2014). The expectations of the effect prescription drugs for cognitive enhancement exert in healthy individuals often exceed their real effect (Repantis et al., 2010). Whether effective or not, the prevalence studies have shown that some people do experiment with drugs to boost their (cognitive) performance (Smith and Farah, 2011).

Herman-Stahl et al. (2007) suggested that the use of prescription drugs to improve one's cognitive performance is especially common among individuals in cognitively demanding environments, such as schools and universities. The authors showed that college students are more likely to use prescription stimulants for nonmedical purposes than young adults who are not enrolled in college, although no intentions of use (e.g., recreational, or to enhance performance) were examined in this study. In addition, most of the prevalence studies conducted so far have focused on the university student population. Smith and Farah (2011) give an overview of 28 epidemiological studies on the prevalence of nonmedical prescription drug use that were conducted among secondary and post-secondary students in the US and Canada before July 2010. The prevalence numbers of lifetime nonmedical stimulant use among university students ranged from 5.3% in an online study reaching over 2000 respondents (DuPont et al., 2008) to 55% in a study among 307 fraternity members (DeSantis et al., 2009). Smith and Farah (2011) conclude that it is hard to draw quantitative conclusions from the list of studies, as they differ in many ways. For example, there are marked differences in the way the prevalence numbers are calculated, where some studies include all nonmedical use, others only include nonmedical use with the intention to enhance ones cognitive performance. This means that in the latter condition users with solely recreational or experimental intentions are excluded from the prevalence numbers that are presented. Moreover, some studies focus solely on the intention, meaning that also individuals with a prescription are counted as a cognitive enhancement drug user when they report to use these drugs for nonmedical reasons, while others exclude all users with a prescription regardless of their intention of use. Smith and Farah (2011) also describe a large variation between studies in sampling method and demographic characteristics, ranging from studies examining a single department at a single institution to nationwide epidemiological surveys on nonmedical drug use. These differences have a major influence on the outcome of the studies because of several factors that can vary between institutions or regions. In a national cross-section study of over 10,000 students enrolled at 4-year colleges McCabe et al. (2005) demonstrated, that the nonmedical use of prescription stimulants was higher in the North-Eastern region of the US, compared to other regions, and higher for institutions with more competitive admission criteria compared to less competitive admission criteria. Finally, a difference can be found in the way that cognitive enhancement drugs are addressed in the questionnaire, for example by either questioning general drug use for the purpose of enhancement or questioning the use of specific types of stimulants (e.g., Ritalin) and/or active ingredient (e.g., methylphenidate) for this purpose. The latter limits the number of stimulants and/or active ingredients that are taken into account in the survey, but leads to more specific questions for the respondent.

Recently more and more studies have been conducted on the university student population in (mostly Western) Europe. The aforementioned variety in definitions and demographic characteristics cause difficulty in comparing the prevalence numbers of these studies as well. For example, Holloway and Bennett (2012) are sometimes mentioned in the discussion on cognitive enhancement drugs, while they focus on general nonmedical use and only briefly mention the intention of cognitive enhancement. From the sample of 1614 students 33% reported to use prescription drugs nonmedical, but only three students reported the reason “to study.” Castaldi et al. (2012) on the other hand, focus solely on the intention, and thus include two students who have a prescription in their prevalence number of 16% of students who use cognitive enhancement drugs in a sample of 77 Italian medical students. Also Pustovrh and Mali (2014) did a (pilot) study in only a single institution, in Slovenia, resulting in a prevalence of 11 out of 211 students (5.21%) who had ever used prescription drugs for cognitive enhancement.

In contrast to the aforementioned studies, the focus of studies seems to shift from prevalence of substance use for cognitive enhancement toward broader topics, such as the motives behind the use, differences between users and non-users, or theoretically grounded accounts of why certain students choose to use drugs for cognitive enhancement while others do not (Eickenhorst et al., 2012; Sattler and Wiegel, 2013; Wolff and Brand, 2013; Wolff et al., 2013, 2014; Ott and Biller-Andorno, 2014). However, prevalence numbers are usually presented as well and will be reviewed here even though they are not always the primary aim of study. Larger studies in Germany and Switzerland demonstrate differences in targeting specific substances or a more general group of pharmacological cognitive enhancers. Sattler and Wiegel (2013), for example, examined prior use by asking whether respondents had ever used a prescription medicine without medical necessity to enhance cognitive efficiency, resulting in a lifetime prevalence of 4.56%. Ott and Biller-Andorno (2014) examined the particular use of Ritalin, Adderall or Modasomil among 1765 students at one Swiss university, and found a prevalence of 6.2% of 1765 students who use prescription drugs without a prescription to increase concentration or alertness, of which 4.7% more specifically reported study purposes. However, they claim the questionnaire is not designed to be representative for their population. Maier et al. (2013) examined the use of 17 specific substances with the intention to enhance cognitive performance, categorized as prescription drugs (e.g., methylphenidate), drugs of abuse (e.g., alcohol) and other substances (e.g., caffeine). They found a prevalence of 7.6% for nonmedical prescription drug use for cognitive enhancement and 7.8% for drugs of abuse for cognitive enhancement among their sample of 6275 students from three Swiss universities. This points to a more widespread phenomenon among several surveys in Germany, to not only include prescription drugs as potential enhancers, but also illicit drugs and/or more commonly available substances (also: lifestyle drugs) such as coffee and energy drinks. Most studies clearly separate them, such as Wolff et al. (2014), who demonstrated the use of lifestyle, prescription and illicit drugs for cognitive enhancement among approximately 1000 students all over Germany, and found a lifetime prevalence of respectively, 83.2, 5.8, and 3.5%. Franke et al. (2011a,b) surveyed the use of coffee, caffeinated drinks, caffeine tablets, prescription drugs, and illicit drugs for cognitive enhancement among high school and undergraduate students and found a prevalence of respectively, 53.2, 39, 10.5, 1.3, and 2.6%. However, other researchers in Germany diverge from this distinction between lifestyle, prescription and illicit drugs, and group them in one general class of cognitive enhancers. Dietz et al. (2013), for instance, report a 12-month prevalence of use of cognitive-enhancing drugs of 20%. In this number the authors include the use of prescription drugs, illicit drugs and caffeine tablets. Eickenhorst et al. (2012) grouped prescription drug use and illicit drug use to improve cognitive performance or mood, and found a prevalence of 7% among 1324 students and graduates.

The current study does not aim to solve the problems that arise due to different definitions, sampling methods or questions, nor do we aim to provide a theoretical framework for pharmacological cognitive enhancement drug use. We aim to contribute to the discussion by examining different study methods in the same sample. This creates the possibility to directly compare the influence of certain questions and inclusion criteria on the resulting prevalence number. Furthermore, the present study sets out to assess the prevalence of pharmacological cognitive enhancement by means of a web-based survey administered to university students in the Netherlands, a sample that to our knowledge has not been examined before. There are only a few studies investigating the use of cognitive enhancers in the Dutch population. Ganpat et al. (2009) surveyed the illicit use of prescription drugs with the intention to improve performance in sport and study among adolescents between 14 and 17 years old and found an overall prevalence rate of 1.7%. A second study assessed the quantity of psychopharmacological enhancement among Dutch psychiatrists and other physicians working in psychiatry (Timmer and Glas, 2012). The results demonstrated a lifetime prevalence of 11%. This latter number is comparable to recent results of a survey among German surgeons (Franke et al., 2013).

In an attempt to reach a representative sample our survey has been advertised online and offline among students of all 14 government supported universities in the Netherlands. The survey examines the prevalence of general nonmedical use of prescription drugs, and the use of particular prescription drugs (methylphenidate, modafinil, beta blockers, and rivastigmine) with the specific purpose of cognitive enhancement with and without a prescription. Furthermore, we will assess the use of prescription drugs for cognitive enhancement with a question not targeted at particular drugs, but at the general group of prescription drugs. Furthermore, we will describe the use of lifestyle and illicit drugs with the purpose of cognitive enhancement. It is impossible to make a comparison of all definitions and study methods in previous studies, but in this way many differences of prior studies are addressed, which makes it possible to directly compare prevalence numbers by different methods within this study and to studies with similar methods abroad.

In addition of giving an overview of different prevalence numbers of the use of substances for cognitive enhancement in the student population in the Netherlands, we also examine two topics that have been found to relate to pharmacological cognitive enhancement, being polydrug use and the relationship between using cognitive enhancement substances and stress. Polydrug use considers the finding that there is a positive relationship between the use of prescription drugs for cognitive enhancement and the use of other substances in general (Barret et al., 2005; McCabe et al., 2005; Eickenhorst et al., 2012; Mazanov et al., 2013). Furthermore, it regards a positive relationship between the use of prescription drugs for cognitive enhancement, the use of illicit drugs for cognitive enhancement and the use of lifestyle drugs for cognitive enhancement, meaning that if somebody uses one of these for cognitive enhancement, there is a higher chance of using substances from the other groups with the purpose of cognitive enhancement as well (Maier et al., 2013; Wolff and Brand, 2013; Ott and Biller-Andorno, 2014). We hypothesize that university students in the Netherlands will also display polydrug use, meaning that (1) users of prescription drugs for the purpose of cognitive enhancement are more likely to use other substances than non-users and (2) there is a correlation between the use of prescription, illicit and lifestyle drugs for cognitive enhancement.

Second, Maier et al. (2013) demonstrated that prescription and illicit drug use for cognitive enhancement is related to perceived pressure to perform. Wolff et al. (2014) found a similar relation for the use of prescription and lifestyle drug use for cognitive enhancement and self-reported strain. Wolff and Brand (2013) demonstrated that overwhelming demands in school could predict the use of prescription drugs and lifestyle drugs for cognitive enhancement. With a longitudinal design Sattler and Wiegel (2013) demonstrated that increased cognitive test anxiety increased the prevalence of nonmedical prescription drug use for cognitive enhancement over time. We hypothesize that a similar relation between the use of cognitive enhancement substances and stress will be prevalent among students in the Netherlands; and thus that students who use substances for the purpose of cognitive enhancement will report more stress than students who do not use substances for the purpose of cognitive enhancement.

Finally, several surveys have examined attitudes toward the use of prescription drugs for cognitive enhancement. In general, the public displays similar concerns as described in the academic literature on the topic, concerning topics such as the safety of using prescription drugs by healthy people, the possibility of being coerced in using a drug for cognitive enhancement and the fairness of using drugs to enhance ones cognitive performance (Schelle et al., 2014). To add to this discussion we assessed opinions in the current sample toward several statements regarding the safety and policy surrounding the use of prescription drugs for cognitive enhancement.

To sum up, the survey aimed at (1) giving an overview of different methods to assess the prevalence of use of substances for cognitive enhancement and apply these methods to a new sample of university students in the Netherlands (2) investigating whether findings of polydrug use and the relationship between cognitive enhancement substance use and stress can also be applied to this population and (3) assessing opinions about the use of prescription drugs for cognitive enhancement.

## Methods

### Respondents

In the current study, 1572 respondents of a total population of approximately 245,000 students registered at Dutch universities, replied to a nationwide poster spread, social media advertisements and a letter to student organizations. Prior to analysis 69 respondents were excluded [exclusion criteria were not being a student at a Dutch university, technical difficulties with the online questionnaire and an affirmative answer on a control question (see below)], resulting in a final sample of 1503 respondents with a mean age of 21.8 years (± sd 2.8 years; 70% women). The sample included students of all 14 government supported Dutch universities, although the relative distribution of respondents was not equal for different universities. Respondents were stimulated to complete the questionnaire by raffling one tablet PC, 30 shopping vouchers (€15,-) and 20 cinema vouchers (€7, 50).

### Procedure

The questionnaire was an anonymous online survey in Dutch, which could only be accessed after signing a digital informed consent form. This form was followed by the questionnaire as described in a next section. After submission of the questionnaire a new non-related website opened where the respondent was offered the opportunity to enter in the lottery for the tablet PC and vouchers. Data were stored in an offline database for later analysis. Care was taken not to store IP addresses from the respondents in the dataset. Contact information needed for distribution of the prizes was stored in a separate data file. The procedure and questionnaire are approved by the Ethics Committee Faculty of Social Sciences (ECSS) of the Radboud University, Nijmegen, the Netherlands.

### Questionnaire

The first section of our questionnaire assessed demographics and background characteristics. Furthermore, we assessed study behavior (e.g., time spent studying) and study outcome to be able to further characterize the cognitive enhancement substance user. After that, we translated and adapted the “Perceived Stress Scale” (Cohen et al., 1983) to a version assessing “Perceived Study Stress” by inserting the word “study” in front of the word “stress” each time this was mentioned in the original scale. The remainder of the survey focused on the use of and the opinion about several substances, the main outcome measures of this study.

The length of the survey depended on the amount of substances used, because respondents were routed toward more specific questions about substances. All questions had a forced response format, meaning that respondents could not skip the question. For most respondents the survey was about 80 questions long. On average, respondents took approximately 18 min to complete the survey.

### Main outcome measures

#### Type of substances

The main outcome measures concern the use of prescription drugs, illicit drugs (stimulants, soft drugs and hard drugs) and lifestyle drugs (alcohol, nicotine, and caffeine). We asked respondents to indicate their use of prescription drugs in two different ways, namely with questions about four prescription drugs in particular (methylphenidate, modafinil, beta blockers, and rivastigmine) and questions about the use of prescription drugs in general.

The use of specific prescription drugs was part of a single question in which we asked respondents to indicate whether they had used one or more of the following substances since the start of their university studies, followed by examples as shown in the following overview: alcohol (e.g., in beer, wine, liquor), nicotine (e.g., in cigarettes, roll-ups), caffeine (e.g., in coffee, energy drinks), pharmacy products (such as painkillers, nutritional supplements), soft drugs (such as marihuana), smart shop products (such as memory boosters, herb pills), stimulants (such as amphetamine, cocaine), hard drugs (such as lysergic acid diethylamide (LSD), heroine), methylphenidate (e.g., in Ritalin), modafinil (e.g., in Provigil), beta blockers (e.g., in Propanolol) and rivastigmine (e.g., in Exelon). We added a non-existent drug to the list of possible substances respondents could indicate to have used. This drug was named “Hoxazine (e.g., in Hypersotaline)” and was placed there to detect untrustworthy results. Respondents who admitted to have used this non-existent drug were excluded from further analysis.

Because we only included four often discussed prescription drugs that could potentially be used for cognitive enhancement we also asked a question regarding general prescription drug use. Respondents were asked to indicate whether they had ever used a prescription drug without having a prescription themselves. If they confirmed this question they were routed toward a question about their reasons for using prescription drugs.

#### Use of substances

If respondents indicated to have been in contact with a substance they were routed toward questions about the specific substance(s) to indicate amount, times and reasons of usage. In the case of prescription drugs they were also asked to indicate whether they had a prescription or not. The answer options for amount, times and reasons of usage were based on questions that have previously been developed for the questionnaire “Family and Health 2003/2004” of the project “Family and Health” of the Radboud University Nijmegen (Heatherton et al., 1991; Engels et al., 1999; Engels and Knibbe, 2000; Harakeh et al., 2005). Specific categories and answer options for amount and times of usage were different for certain substance categories. Therefore, they will be discussed in the following sections regarding the specific substances. Answer options for amounts and times of usage of substances which were not present in the “Family and Health 2003/2004” were created based on questions asked on a similar substance in the questionnaire.

The “Family and Health 2003/2004” questionnaire discusses four general reasons for substance usage: coping with stress, conformity to peers, enhancement (feeling good) and social substance use, related to the four-factor model of alcohol use (Cooper, 1994). We added four answer options in the question about reasons for usage for each substance: (1) “to enhance study performance” as this was our main target of interest, (2) “medical reasons” and (3) “to lose weight” as it applied to some of the substances different than alcohol on which the four-factor model was based, and (4) “other” to provide respondents with an answer option in case their answer was not in the list.

From these questions specific measures per substance (category) were derived. The amount of nonmedical users was calculated by including all users of the substance (category) who reported to at least sometimes (on a four-point scale of seldom; sometimes; regularly; often) use the substance for any other than “medical reasons” (exception: general prescription drug use, for which this measure could only be calculated for users who do not have a prescription themselves). The amount of users using a substance (category) without a prescription was calculated by including all users of the substance (category) minus the users who reported to use the substance without “regularly” or “often” (similar four-point scale of seldom; sometimes; regularly; often) to have a prescription. The amount of users using the substance (category) with the purpose of cognitive enhancement was calculated by including all users of the substance who reported to use the substance at least sometimes “to enhance study performance” (similar four-point scale).

#### Opinions: prescription drug use for cognitive enhancement

Further, we were interested in the attitude of our respondents toward the use of prescription drugs for the purpose of cognitive enhancement. Respondents were presented with 17 statements and consequently asked to provide a response on a five-point Likert scale, indicating their (dis) agreement with the statement at hand (see **Table 2** for the list of statements). Before reading these statements we presented a short description of the term “smart pills” to the respondents (see Box 1 for a translated version of the Dutch text).

Box 1 **Description of the term “smart pills” (translated from Dutch).**

Some students use drugs which are only available with a prescription, without having a prescription themselves. Some other students do have a prescription, but they use a higher dose than prescribed. When they use those drugs to improve their study results, these drugs are called smart pills. The statements below are all about smart pills. Please fill in to what extent you agree with these statements.

### Data analysis

The first hypothesis, regarding polydrug use, has two components. Chi-square analyses were conducted to examine whether users of prescription drugs are more or less likely to use other substances than non-users. Secondly, Chi-square analyses were conducted to examine the association between the use of prescription drugs, illicit drugs, and lifestyle drugs for the purpose of cognitive enhancement. The second hypothesis, regarding the relationship between substance use for cognitive enhancement and stress, was examined with One-Way ANOVA's for the differences in perceived study stress scores between users and non-users according to our different operationalizations. *P*-values < 0.05 (two-tailed) were considered statistically significant. The analyses were conducted with IBM SPSS Statistics version 22.0.

## Results

### Prescription drug use

#### Use of specific prescription drugs

None of the respondents reported to use rivastigmine or modafinil. In our sample, 52 students indicated to have used methylphenidate, while 36 students indicated to have used beta blockers. Nonmedical use (at least “sometimes” use for any other reason than medical) of methylphenidate was self-reported by 80.8% of methylphenidate users (2.8% of the total sample of respondents) and 61.1% of beta blockers users (1.5% of the total sample of respondents). Total nonmedical use of specific prescription drugs in the sample is 4.0% (due to four users using both beta blockers and methylphenidate at least sometimes for nonmedical purposes). Excluding the respondents who indicated to “regularly” or “often have a prescription” results in 2.4% of respondents reporting to use prescription drugs at least sometimes for a nonmedical purpose without having prescription.

From the methylphenidate users 73.1% (2.5% of the total sample of respondents) reported to use methylphenidate at least sometimes for the specific purposes of improving ones study results (cognitive enhancement). From the beta blocker users 38.9% (0.9% of the total sample of respondents) reported to use beta blockers at least sometimes for the specific purpose of improving ones study results. Total specific prescription drug use with the intention of cognitive enhancement in the sample is 3.2%.

Furthermore, to examine the prevalence of the use of prescription drugs for the purpose of cognitive enhancement without having a prescription, we excluded respondents who indicated to use prescription drugs regularly or often with a prescription. A group of 40.4% of the methylphenidate users reported to use methylphenidate less than often with a prescription, and at least sometimes for the purpose of cognitive enhancement (1.4% of the total sample of respondents). For beta blockers this prevalence was 19.4% (0.5% of the total sample of respondents). Total use of specific prescription drugs with (at least sometimes) the intention of cognitive enhancement without (regularly or often) having a prescription is 1.7%. Table 1 shows via which other methods these respondents obtained their prescription drug. Most users without a prescription acquire the drugs via people they know.

**Table 1:**
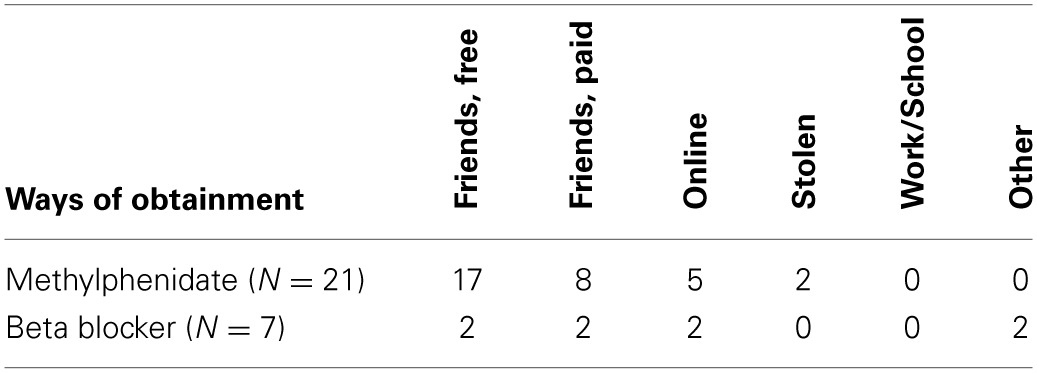
**The number of respondents using specific routes of obtainment for prescription drugs with the purpose of cognitive enhancement from the group of respondents who indicated to “less than often” have a prescription for the drug themselves**.

#### Use of a general group of prescription drugs

Sixty respondents (4.0% of the total sample of respondents) admitted to use a prescription drug without having a prescription for the drug. Forty-six respondents (3.1% of the total sample of respondents) reported nonmedical use without a prescription (at least “sometimes” use for any other reason than medical). From the 60 respondents, 40% used these prescription drugs at least sometimes to improve their study results (2.7% of the total sample of respondents).

#### Opinions about prescriptions drug use for cognitive enhancement

The percentage of respondents that (dis)agreed with 17 statements about the use of “smart pills” are presented in Table 2. Most respondents disagree with statements regarding the respondent being aware of the use of smart pills. There is less agreement about statements regarding the risks related to the use of smart pills, the fairness of the use of smart pills, and the regulation of the use of smart pills.

**Table 2:**
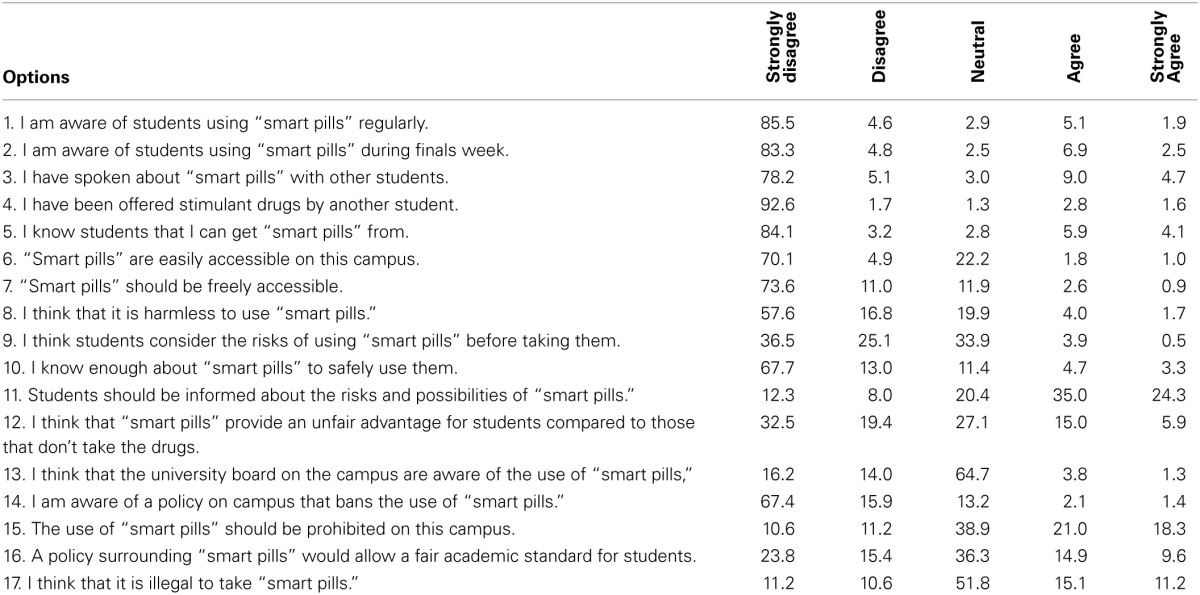
**Percentages of agreement toward statements about smart pills (*N* = 1503)**.

### Illicit drug use

Soft drugs, hard drugs and stimulants are considered illicit drugs. Table 3 displays the prevalence of the use of the three categories of illicit drugs, the use of illicit drugs for nonmedical reasons (at least “sometimes” use for any other reason than medical) and the use of illicit drugs with the specific purpose of cognitive enhancement. Total use of illicit drugs is 20.5%. The use of illicit drugs for nonmedical reasons is 20.4% and with the specific purpose of cognitive enhancement (CE) 1.3%.

**Table 3:** **Prevalence of the use of soft drugs, hard drugs and stimulants, calculated from the total sample**.

	**Soft drugs (%)**	**Hard drugs (%)**	**Stimulants (%)**
Use	19.1	1.9	4.9
Nonmedical use	19.0	1.9	4.8
Use for CE	0.7	0.1	0.6

### Lifestyle drug use

Alcohol, nicotine, caffeine, over the counter pharmacy products, and (legal) smart shop products are considered lifestyle drugs. Table 4 displays the prevalence of the use of the categories of lifestyle drugs, the use of lifestyle drugs for nonmedical reasons (at least “sometimes” use for any other reason than medical) and the use of lifestyle drugs with the specific purpose of cognitive enhancement. Total use of lifestyle drugs is 92.8%. The use of lifestyle drugs for nonmedical reasons is 90.5% and with the specific purpose of cognitive enhancement 45.6%.

**Table 4:** **Prevalence of the use of alcohol, nicotine, caffeine, over the counter pharmacy products and (legal) smart shop products, calculated from the total sample**.

	**Alcohol (%)**	**Nicotine (%)**	**Caffeine (%)**	**Pharmacy (%)**	**Smart shop (%)**
Use	84.3	20.3	69.1	59.6	3.7
Nonmedical use	83.2	19.8	66.2	36.8	3.2
Use for CE	1.8	3.0	41.7	9.0	0.5

Table 5 provides an overview of the prevalence of the use of certain drugs according to the above described definitions.

**Table 5:** **An overview of the prevalence of the use of drugs for CE**.

Nonmedical use of specific prescription drugs with/without prescription	4.0%
Nonmedical use of specific prescription drugs without a prescription	2.4%
Use of specific prescription drugs with/without prescription with the intention of CE	3.2%
Use of specific prescription drugs without a prescription with the intention of CE	1.7%
Nonmedical use of general group of prescription drugs without a prescription	3.1%
Use of general group of prescription drugs without a prescription with the intention of CE	1.6%
Nonmedical use of illicit drug	20.4%
Use of illicit drugs with the intention of CE	1.3%
Nonmedical use of lifestyle drugs	90.5%
Use of lifestyle drugs with the intention of CE	45.6%

### Polydrug use

Users of specific prescription drugs with/without a prescription reported more often the use of soft drugs, stimulants, nicotine and over the counter pharmacy products. Users of prescription drugs without a prescription were more likely than non-users to report use of soft drugs, nicotine, caffeine and over the counter pharmacy drugs. Table 6 provides the test statistics for these tests. For most substances the data confirm the hypothesis that users of prescription drugs for the purpose of cognitive enhancement are more likely to use other substances than non-users of prescription drugs for the purpose of cognitive enhancement.

**Table 6:** **Results of the Chi square comparisons of substance use between users and nonusers of prescription drugs, with/without and only without a prescription**.

	With and without a prescription (*N* = 48)		without a prescription (*N* = 25)	
	**χ^2^**	**Odds ratio**	**χ^2^**	**Odds ratio**
Alcohol	2.03		2.36	
Nicotine	30.98^**^	4.56	35.77^**^	8.78
Caffeine	3.41		4.24^*^	3.32
Pharmacy	7.87^**^	2.64	6.28^*^	3.62
Soft drugs	13.47^**^	2.90	22.41^**^	5.62
Smart shop products	X		X	
Stimulant drugs	20.71^**^	5.02	X	
Hard drugs	X		X	

Significance levels are indicated with

* P < 0.05 and

** P < 0.01. X is used to indicate that a statistical test was not performed as small cell sizes precluded significance testing.

Furthermore, Chi square tests of the association between prescription drugs, illicit drugs, and lifestyle drugs for the purpose of cognitive enhancement demonstrate that users of lifestyle drugs for the purpose of cognitive enhancement are more likely to use prescription drugs and illicit drugs for the purpose of cognitive enhancement. This partly confirms the hypothesis that there is an association between the use of prescription, illicit, and lifestyle drugs for cognitive enhancement. Table 7 provides the test statistics for these tests. Similar tests for the relation between the use of illicit drugs for the purpose of cognitive enhancement and prescription drugs for the purpose of cognitive enhancement could not be performed because small cell sizes precluded significance testing.

**Table 7:** **Results of the Chi square association between the use of prescription drugs (specific prescription drugs with/without a prescription; specific prescription drugs without a prescription; general prescription drugs without a prescription), illicit and lifestyle drugs for cognitive enhancement**.

	**Specific with/without**		**Specific without**		**General without**		**Illicit**	
	**χ^2^**	**OR**	**χ^2^**	**OR**	**χ^2^**	**OR**	**χ^2^**	**OR**
Lifestyle	22.56^**^	4.75	12.15^**^	4.89	8.51^**^	3.65	9.68^**^	4.87

Significance levels are indicated with ^*^P < 0.05 and

** P < 0.01. The odds ratio (OR) is calculated as a measure of the effect size.

### Cognitive enhancement and stress

Users of specific prescription drugs with/without a prescription for the purpose of cognitive enhancement and users of lifestyle drugs for the purpose of cognitive enhancement report more study related stress than their respective non-user groups. This means that our hypothesis that students who use substances for the purpose of cognitive enhancement will report more stress than students who do not use substances for the purpose of cognitive enhancement is only confirmed for certain definitions of cognitive enhancement drug use. Table 8 provides the according test statistics.

**Table 8:** **Results of One-Way ANOVAs for the relation between the use of prescription drugs (specific prescription drugs with/without a prescription; specific prescription drugs without a prescription; general prescription drugs without a prescription), illicit and lifestyle drugs for cognitive enhancement and study related stress**.

	**Users M (sd)**	**Nonusers M (sd)**	**F (df 1,1501)**	***r***
Specific w/wo	39.29 (7.74)	36.67 (6.81)	35.75^*^	0.067
Specific wo	38.68 (8.61)	36.72 (6.82)	2.02	
General wo	38.25 (8.56)	36.73 (6.82)	1.17	
Illicit	38.00 (6.61)	36.73 (6.86)	0.67	
Lifestyle	37.89 (6.99)	35.79 (6.59)	35.75^**^	0.153

Significance levels are indicated with

* P < 0.05 and

** P < 0.01.

## Discussion

The present study demonstrates the prevalence of the nonmedical use and the use with the specific purpose of cognitive enhancement of prescription drugs, illicit drugs, and lifestyle drugs among university students in the Netherlands. General use and nonmedical use is larger for lifestyle drugs than illicit drugs, and larger for illicit drugs than prescription drugs. Even though prevalence numbers differ for general and nonmedical use, the use of prescription drugs and illicit drugs with the purpose of cognitive enhancement is rather similar and low in occurrence in the current sample. However, almost half of the respondents use lifestyle drugs with the intention to cognitively enhance themselves. Users of prescription drugs for the purpose of cognitive enhancement are more likely to use other substances than non-users. There is a relation between the use of prescription drugs and the use of illicit drugs, but not with the use of lifestyle drugs when it comes to using these substances to cognitively enhance oneself. The hypothesis, that respondents who use substances for cognitive enhancement experience more stress than non-users, is confirmed for the group of users of specific prescription drugs that include both users with and without a prescription and for users of lifestyle drugs.

The use of substances for cognitive enhancement has not previously been examined in the university student population in the Netherlands. However, the prevalence of the use of prescription drugs without a prescription for the purpose of cognitive enhancement, is in line with a study within a younger Dutch population (14–17 year) in which 1.7% reported to use prescription drugs—which were not prescribed to themselves or in a different way than prescribed to them—with the intention the enhance performance (Ganpat et al., 2009). Compared to the nonmedical use of prescription drugs in Wales, our result of nonmedical use is far lower (Holloway and Bennett, 2012). The same trend is observed for the use of substances for the specific purpose of cognitive enhancement. The use of specific prescription drugs and general prescription drugs with this intention (1.7 and 1.6%) is lower than most lifetime prevalence numbers in Europe (ranging from 4.6 to 16%) (Castaldi et al., 2012; Maier et al., 2013; Sattler and Wiegel, 2013; Ott and Biller-Andorno, 2014; Pustovrh and Mali, 2014; Wolff et al., 2014). It might be suggested that our numbers are lower due to their timeframe being based on “during respondent's university studies” instead of lifetime prevalence. However, Eickenhorst et al. (2012) also examined use “during studies” and found a prevalence of 7%, similar to other lifetime reports. Furthermore, the only two studies with a low prevalence more similar to our prevalence are conducted in Germany by Franke et al. (2011a) with a lifetime prevalence of 0.8% and by Mache et al. (2012) with a lifetime prevalence up to 2%.

The use of illicit drugs to enhance cognitive performance in our sample (1.3%) is a little lower than previous prevalence numbers of 2.9% (Franke et al., 2011a) and 3.5% in Germany (Wolff et al., 2014; however, a smaller difference is found when comparing to their finding of the point prevalence of 1.7%). When we combine our prevalence of the use of illicit drugs and alcohol for cognitive enhancement and compare it with the category of drugs of abuse (7.8%) by Maier et al. (2013), the resulting prevalence of our study is clearly lower. The use of lifestyle drugs to enhance cognitive performance (45.6%) is lower than found by Wolff et al. (2014; 83.2%), but more in line with the prevalence of the use of coffee for this purpose by 53% respondents of Franke et al. (2011a) and what the authors report as soft enhancers (e.g., coffee) by about half of the respondents of Maier et al. (2013). Again, when looking at the point prevalence instead of lifetime prevalence of the use of lifestyle drugs for cognitive enhancement (52.3%) by Wolff et al. (2014), the difference is smaller. In general our prevalence numbers of prescription drugs and illicit drugs appear to be low compared to research in other European countries, whilst our results for lifestyle drugs are more in line.

One potential reason that explains the low prevalence of the use of substances for cognitive enhancement among university students in the Netherlands can be found by taking a closer look at responses to statements regarding the awareness about the use of prescription drugs for cognitive enhancement purposes. Whereas in a Swiss sample 93.7% of the respondents and in a German sample almost 60% knew that substances could and are being used for the purpose of cognitive enhancement, only 13.7% of our current sample has ever spoken about the use of prescription drugs for cognitive enhancement (Franke et al., 2011a; Maier et al., 2013). Lesser awareness of the possibility to use substances for the purpose of cognitive enhancement among peers can have an impact on the prevalence, in particular for prescription drugs which, when not obtained by a prescription, are often obtained via peers. This is in line with previous research (McCabe and Boyd, 2005; Maier et al., 2013; Ott and Biller-Andorno, 2014).

Although awareness of the use of cognitive enhancement drugs by others is rather low (10%), it is still considerably higher than the actual prevalence of the use of prescription drugs for cognitive enhancement (1.6–3.2%). It remains unclear whether this difference is based on the fact that respondents know the same users, whether students overestimate the use of prescription drugs for cognitive enhancement by other students, whether there is a bias in sampling toward more non-users, or whether they did not report their own use truthfully. Recent research suggests that prevalence rates vary depending on the type of questions. Previous research by Franke et al. (2011a) showed a prevalence in Germany of below 1% while a more recent study, using an altered version of the Randomized Response Technique estimated a prevalence of 20% prescription drugs use for cognitive enhancement in the German student population (Dietz et al., 2013). Dietz et al. (2013) relates this difference to the stigmatized nature of the topic of enhancement drug use, which would indicate that our results demonstrate an underestimated prevalence rate due to non-truthful answers. However, other researchers emphasize the overestimation of the use of cognitive enhancement drugs by peers, which would suggest that the number of 9%, of students who know other students to be users, is unreliable, while our actual prevalence rate is more reliable (Lucke et al., 2011).

In spite of the low prevalence numbers, the majority of the students believe they ought to be receiving adequate information about the risks and opportunities of using prescription drugs for cognitive enhancement. This is in line with findings from previous studies on the opinions about pharmacological cognitive enhancement in which people argued that it was important to be able to make autonomous decisions about the use of such substances (Schelle et al., 2014). However, deciding whether or not to give students or the general population more information about the use of substances for cognitive enhancement is a difficult task. Sandberg (2013) argues that on the one hand the lack of information can create irrational demand and employer coercion when hype will dominate, but at the same time there is not enough information yet—especially on the long term effects—to create a proper risk/benefit analysis.

Modafinil and rivastigmine were not reported as being used by the students in our sample, while being proposed as two of four specific prescription drugs that could be used for cognitive enhancement. One explanation for this discrepancy could be that these two drugs are not as often prescribed in the Netherlands to students compared to methylphenidate and beta blockers. For example, while modafinil is only 10,500 times provided by the public pharmacies in the Netherlands in 2009, the amount of times methylphenidate was provided was already rising to a million prescriptions per year, which it passed in 2011 (Stichting Farmaceutische Kengetallen, 2010, 2012). In contrast, rivastigmine is often prescribed to older people, which may lead to its accessibility being scarce in the student population, especially when compared to the amount of methylphenidate prescriptions in the population. This explanation based on the amount of prescriptions in the corresponding age population is supported by the fact that most prescription drugs which are not obtained by a prescription are obtained via peers, either freely or paid. This corroborates previous studies in other countries (McCabe and Boyd, 2005). It would be interesting to further examine whether different populations in the Netherlands do use prescription drugs like rivastigmine and modafinil for the purpose of cognitive enhancement, especially focusing on those populations for whom the specific effect of the substance is more desirable, or the substance is more accessible. Examples of specific target populations are academics, pilots and surgeons (see e.g., Franke et al., 2013).

As hypothesized, users of prescription drugs for cognitive enhancement reported more often the use of other substances such as soft drugs, nicotine and stimulants than non-users. This polydrug use is consistent with previous studies (Barret et al., 2005; McCabe et al., 2005; Teter et al., 2006; Eickenhorst et al., 2012; Mazanov et al., 2013). Polydrug use is also found in other domains, such as doping in sport (Dunn et al., 2009; Backhouse et al., 2013). Based on their co-occurrence Backhouse et al. (2013) suggest that athletes who engage in legal performance enhancement are an “at-risk” group for transition toward doping, the gateway hypothesis. Our findings display that users of lifestyle drugs for the purpose of cognitive enhancement are more likely to use illicit drugs and prescription drugs for the purpose of cognitive enhancement and thus support previous findings in suggesting that this hypothesis not only plays a role in physical performance enhancement but also for cognitive performance enhancement.

Furthermore, in accordance with previous studies there is an association between the use of prescription drugs and illicit drugs for cognitive enhancement purposes. In contrast to previous findings, no such relation was found between the use of prescription drugs and lifestyle drugs for cognitive enhancement purposes (Maier et al., 2013; Wolff and Brand, 2013; Ott and Biller-Andorno, 2014). An explanation of this discrepancy can be found in the way that the category of lifestyle drugs is approached and measured. Ott and Biller-Andorno (2014) found that use of cigarettes could explain variance in their logistic regression model about cognitive enhancement drug use, while coffee did not. Another explanation might be found in the low awareness about the use of prescription drugs for cognitive enhancement in the current sample. There might be a higher discrepancy between the awareness of the use of prescription drugs for cognitive enhancement and the use of lifestyle drugs for cognitive enhancement than in other countries, resulting in less association between the two types of cognitive enhancement drug use.

Finally, it was proposed that students who use substances for the purpose of cognitive enhancement report more stress than non-users. This hypothesis is confirmed for the group of users of specific prescription drugs that include users with and without a prescription, and for the group of users of lifestyle drugs for cognitive enhancement. Lifestyle drugs are most commonly used for cognitive enhancement in our sample, by almost half of all respondents. Our findings suggest that stress might be a predicting factor for using lifestyle drugs for cognitive enhancement. A similar longitudinal study as Sattler and Wiegel (2013) conducted on the relation between cognitive test anxiety and the use of prescription drugs for cognitive enhancement would inform whether the use of lifestyle drug use is a coping mechanism for (study related) stress or a potential cause of stress. The finding that there is no relation between the use of illicit drugs for cognitive enhancement and stress is supported by previous findings of Wolff et al. (2014) and Wolff and Brand (2013) but not Maier et al. (2013). More research is needed to explore these contrasting findings. Furthermore, future research can also give more insight in why students in the Netherlands who indicate to use a general group of prescription drugs for cognitive enhancement, or specific prescription drugs without having a prescription, do not confirm the previous associations between prescription drugs use for cognitive enhancement and stress (Maier et al., 2013; Sattler and Wiegel, 2013; Wolff et al., 2014).

In this study, a differentiation between different operationalizations of cognitive enhancement substance use is presented. By displaying results based on different operationalizations we aim to create the possibility to compare our results with as many studies as possible and inform the debate about differences in the result due to different research methods. As several of the prevalence numbers of the use of substances for cognitive enhancement in our sample are rather low, large differences between the operationalizations have not arisen. However, our findings suggest that there is less influence of asking about specific prescription drugs vs. a general kind of prescription drugs than there is influence of including or not including users with a prescription for a prescription drug. Furthermore, our findings demonstrate a large difference between asking nonmedical use of any substance or asking about use for the specific purpose of cognitive enhancement. This supports the importance of a means-to-end relation as proposed by Wolff and Brand (2013) and Wolff et al. (2014).

In addition to the different operationalizations that were proposed in an attempt to tackle most differences between studies on the prevalence of the use of substances for cognitive enhancement, a further topic of interest is found. Recently, more and more researchers start including not only the use of prescription drugs, but also the use of illicit and lifestyle drugs in the debate about cognitive enhancement. It was hard to define which substances belong to which overarching categories, as several frameworks can be chosen to underlie these categories (e.g., legal; normative acceptance; accessibility). Therefore, different categories are used, such as lifestyle drugs vs. soft enhancers (Maier et al., 2013; Wolff et al., 2014) and illicit drugs vs. drugs of abuse (Franke et al., 2011a; Maier et al., 2013; Wolff et al., 2014). When one wants to relate the use of a certain category of substances to for example stress, it is clear that these relations depend on the type of substances included in a certain category. We aimed to create transparency about this topic by providing the prevalence number for each specific substance within each category separately.

### Limitations

A limitation to this study is the sample which, due to convenience sampling, constituted only an approximate representation of the student population in the Netherlands. Women, for example, were oversampled. In addition, the sample was not equally distributed for different universities, as well as not distributed in line with the absolute difference in amount of students of the 14 Dutch government supported universities. However, the percentages of recreational substance use obtained in our sample are lower, but within normal limits, than numbers specified in the National Drug Monitor in the Netherlands (Van Laar et al., 2012). This indicates that, regarding our main topic of drug use, our sample is representative for the population. Therefore, no weighting strategies are applied.

Furthermore, it is clear that although the use of substances for cognitive enhancement is mostly examined in student populations, it is probably not possible to generalize this to other populations. This is supported by the prevalence rate of prescription drug use for cognitive enhancement of 11% among Dutch psychiatrists, which is rather high compared to the prevalence in the current sample (Timmer and Glas, 2012). Even similar aged non-student populations may demonstrate a different pattern of use (Herman-Stahl et al., 2007). Future studies will need to give more insight in the use of substances for cognitive enhancement in other target groups such as in specific occupations, or specific age groups, both within the Netherlands as abroad.

A final limitation regards the study methods. The research is conducted by an online large-scale self-report questionnaire with many questions which might have given rise to a decrease of the feeling of anonymity, a burden in the time that was needed to complete the questionnaire, or for example a lack of memory about certain situations and feelings during the use of certain substances. It is possible that the use of certain substances is stigmatized (Dietz et al., 2013), which may lead to a different reported prevalence rate compared to the actual prevalence rates.

## Conclusion

To sum up, the present study indicates that the use of prescription drugs and illicit drugs to increase cognitive performance among students in Dutch universities is rather low compared to other European countries, while the use of lifestyle drugs for cognitive enhancement fits the European context better. We have found further evidence of polydrug use in relation to cognitive enhancement, while previous findings of the relation between cognitive enhancement drug use and stress have not been confirmed consistently. We have decided to report the findings of several operationalizations of cognitive enhancement drug use to enable comparison with a wider variety of previous and upcoming research. We urge future researchers to take the discussion about these different operationalizations and the effects that they have on the prevalence numbers into account in designing and reporting future experiments on the use of substances for cognitive enhancement.

### Conflict of interest statement

The authors declare that the research was conducted in the absence of any commercial or financial relationships that could be construed as a potential conflict of interest.
